# Effects of prenatal antibiotic treatment on early infant health: a retrospective study in a rural health facility in Ghana

**DOI:** 10.4314/ahs.v23i1.44

**Published:** 2023-03

**Authors:** Kwame Opoku-Agyeman, Paa Kofi Tawiah Adu-Gyamfi, Charles Ansah, Kwesi Boadu Mensah

**Affiliations:** 1 Department of Pharmacy, Seventh-Day Adventist Hospital-Kwadaso; 2 Department of Pharmacology, College of Health Sciences, Kwame Nkrumah University of Science and Technology, Kumasi, Ghana; 3 Department of Nursing and Midwifery, Pentecost University, Accra, Ghana

**Keywords:** Intrapartum antibiotic, microbiome, sub-Sahara Africa, neonatal sepsis

## Abstract

**Background:**

Infant mortality remains a major developmental challenge in many low-income countries. Epidemiological evidence suggests that infant acquisition of maternal microbiome is essential for programming of immunity and metabolism. As such, irrational maternal antibiotic use may affect infant health.

**Objectives:**

The aim of the study was to determine the effects of prenatal antibiotic use on early postnatal life (90 days) in a low-income community in Ghana.

**Methodology:**

The study was a retrospective study of 412 mother-baby pair medical records in a low-income community in rural Ghana.

**Results:**

During the ninety-day period, the prevalence and relative risk of neonatal sepsis, respiratory disorders, and dermatitis were significantly higher in infants treated prenatally with antibiotics compared to untreated infants. Prenatal antibiotic treatment was not significantly associated with the risk of developing neonatal jaundice and conjunctivitis. However, prenatally antibiotic exposed infants were three times likely to visit the hospital for a non-scheduled/non-review treatment within the first 90 days compared to unexposed babies.

**Conclusions:**

Intrapartum antibiotic treatment is associated with poor early infant health. Rationalizing antibiotic use during pregnancy may contribute to reducing infant mortality.

## Introduction

Although there has been a steady decline in recent years, maternal and infant mortality remain a major developmental challenge in many low-income countries^1^. Globally, 295,000 women died during or immediately after child birth in 2017. Approximately 94% of these maternal deaths occurred in low-income countries. Two-thirds of these deaths occurred in sub-Sahara Africa^1^. In Ghana, although maternal mortality declined by 37% between 2000 –2017, it still remains high at 211 deaths per 100,000 live births^1^. During pregnancy, the maternal immune system adapts to accommodate the conceptus, which may make her susceptible to some infectious diseases.^2^–^3^. Consequently, it has been shown that pregnant women show reduced immune response to infections and the course of several autoimmune disorders is altered 4–5. The role of estradiol and progesterone in modulating maternal immunity during pregnancy has been demonstrated elsewhere^4^. The foetus also develops characteristics that promote its assimilation into the mother. It is known that it exhausts cellular essential amino acid, tryptophan, which is essential for T- cell proliferation 6–7.

To protect both maternal and foetal health, several anti-infective medicines are employed. In many sub-Saharan Africa countries, very few laboratories are equipped to isolate and identify causative organisms often leading to irrational antibiotic prescription patterns in pregnancy resulting in possible foetal exposure to antibiotics in utero 8,9. We had earlier reported that 65% of pregnant women in a rural community in Ghana were treated with antibiotics at some point during pregnancy 10. Indeed, antibiotic use within 24 hours of delivery was associated with decreased mean scores for appearance, pulse, grimace, activity, and respiration (APGAR) 10. This effect was independent on the class or type of antibiotic used. It is also known that third trimester and early postnatal period are very critical for programming of infant immunity and metabolism 11–12. It has been shown that microorganisms acquired by a baby from the mother's birth canal during delivery may play an essential role in the programming of the immunity and metabolism of the baby 13–14. This presupposes that treating mothers during pregnancy with antibiotics may alter the kind and quality of microorganisms passed on to the babies; making the babies susceptible to immunologic and allergic diseases in later life. Indeed, several studies have reported a positive association between the quality of acquired maternal microbiome and the incidence of neonatal respiratory tract diseases and neonatal sepsis 15–17.

Infectious diseases are a leading cause of early infant mortality. A compromised immunity predisposes the infant to infectious diseases. Unfortunately, over 99% of infant mortality occur in areas where less than 1% of research into infant mortality is carried out 18. To address the high incidence of infant mortality in sub-Saharan Africa, we had hypothesized that intrapartum antibiotic usage may be associated with poor infant health in the early neonatal period. The aim of the study was to determine the effects of prenatal antibiotic use on early postnatal life (90 days) in a low-income community in Ghana. This study is a retrospective follow-up study into the health status of children born to mothers who were treated with antibiotics during pregnancy.

## Method

### Study area

The study site, National Health Insurance, is located within the Bekwai Municipality near the Greater Kumasi Area of the Ashanti Region of Ghana. The area is rural and the majority of inhabitants engage in peasant farming and/or petty trading. The hospital is a 45-bed capacity district hospital. It runs an ante-natal and post-natal clinic. It has a resident Gynaecologist, Pharmacist, several Midwives and Nurses. Most patients access health through the capitation program of the National Health Insurance. The area was chosen because it was rural with no pharmacy and many of the people used National health insurance, this was supposed to help check out of hospital antibiotic usage which is a major confounding factor for the study.

### Study design, sampling and Sample size determination

Based on the fertility rate of the municipality (110.5 per 1000 women) and applying Cochran (1977) equation, a minimum of 325 respondents were required 19–20. The study period was January 2011 to December 2015. Five hundred (500) mother/baby pair folders were randomly selected for each of the study years from the hospitals maternity folder database. However, only 2100 folders could be retrieved from the Biostatic and records unit. Applying the inclusion criteria, only 412 folders were included in the study.

**The Inclusion criteria were as follows:**

- Mother should have attended at least three Antenatal Clinics, delivered at the hospital and attended postnatal clinic at the facility.
- The baby should have attended all scheduled reviews

**The exclusion criteria were as follows:**

- Mother-baby pairs with no records or scanty postnatal records
- Babies who were not breastfed or who missed scheduled review visits
- Twin births, births by referrals were also excluded.

### Definitions

- In this study, impetigo, dermatitis and eczema were all categorized as dermatitis.
- Rhinitis, chest infection, pneumonia, bronchiolitis and any other condition related to the respiratory tract were accepted us Respiratory tract disease.
- The mean number of hospital visits was obtained using the count of the number of times the child was brought to the hospital to seek medical treatment or attention. Normal post-natal review visits or scheduled visits were excluded from the count of visits.
- The term “neonatal conjunctivitis” was chosen to represent any diagnosis of a prescriber listed as follows; neonatal conjunctivitis, septic or bacterial conjunctivitis, allergic conjunctivitis and ophthalmia neonatorum.

### Ethics approval

Ethical approval for the study was given by the Committee on Human Research, Publications and Ethics, Kwame Nkrumah University of Science and Technology, School of Medical Sciences and Komfo Anokye Teaching Hospital, Kumasi (CHRPE/AP/347/16). Permission to carry out the research was given by the hospital management committee of Seventh-Day Adventist Hospital, Dominase (ref; RDO1/16). Individual permission and consent were not possible as mother/baby pair medical records were the principal source of data. For confidentiality, patient's name and address were also excluded.

### Statistical analysis

Data from mother/baby pair folders were first captured with a special questionnaire and transferred unto IBM Statistical Package for Social Sciences (SPSS) version 21 for analysis. Categorical variables such as the presence or absence of a neonatal condition were analysed with Pearson Chi-square. P value less than or equal to 0.05 at 95% confidence interval was considered statistically significant. Relative risk was used as an indicator of the degree of association between a condition and comparable groups.

### Limitations of the study and managing potential confounders

The choice of low-income deprived rural community without a community pharmacy limited the impact of potential out-of-hospital antibiotic use. Secondly, the “capitation policy” of the National Health Insurance Scheme of Ghana encouraged mother/baby to seek health at the hospital instead of elsewhere where they have to pay out of pocket. This made respondents more likely to seek treatment from the hospital. Nonetheless, the study cannot completely rule out the possibility of some out-of-hospital antibiotic use. The study could not account for the effects of diet, herbs sometimes used for lactation on the study outcome 21. The study could not fully account for the effects of maternal health during pregnancy on early postnatal infant health.

## Results

### Study Population characteristics

**Figure 1:**
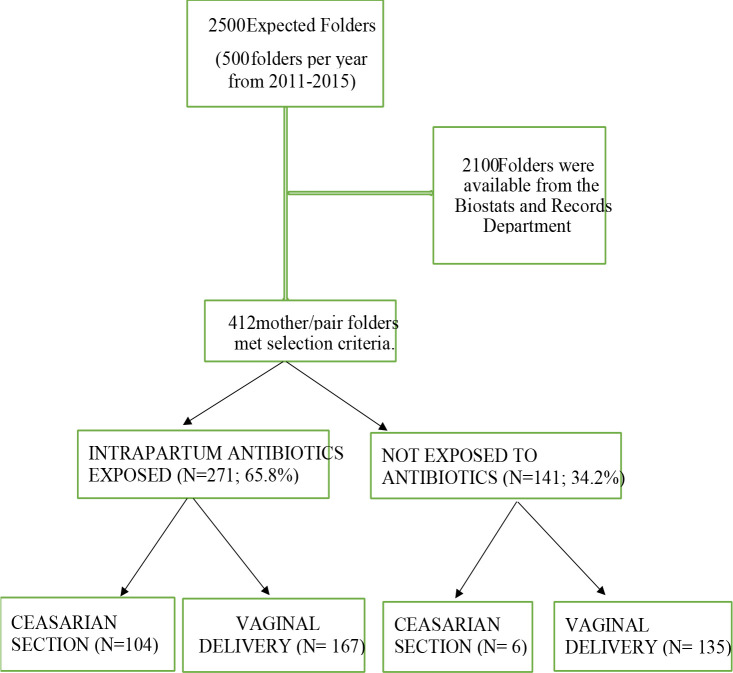
Diagram showing a summary of Mother/Baby pair folder sampling, Mode of delivery and antibiotic use during pregnancy. There was a significant association between the mode of delivery and prenatal use of antibiotics (P<0.001, K=55.47, DF=1 OR =13.8)

In this study, the health of infants whose mothers were treated with antibiotic during pregnancy (intrapartum antibiotic exposed) was compared with infants whose mothers were not treated with antibiotics during pregnancy. Four hundred and twelve (412) mother-baby pairs met the inclusion criteria for the study. Of the 412 infants involved in the study, 271 children were exposed to intrapartum antibiotics at some stage of pregnancy. One hundred and forty-one (141) infants who were not exposed to intrapartum antibiotics served as controls (Figure 1). Three hundred and two infants (302) were delivered vaginally (natural delivery) and one hundred and ten (110) were delivered through Caesarean section. There was a strong association between prenatal antibiotic use and mode of delivery (Odd ratio =13.8, *P*<0.001, K=55.47, DF=1). Fifty-five percent (55%) of babies delivered vaginally were treated prenatally with antibiotics, whereas 94.5% of babies who born by Caesarean were exposed to antibiotics intrapartum (Figure 1)

### Health of Neonates at the Beginning of the Study (Postnatal Day Zero).

There was no statistically significant difference in terms of mean birth weight, mean APGAR scores, birth defects (malformations) between neonates exposed to intrapartum antibiotics and unexposed neonates at birth (Day 0). There was also no statistically significant difference in mean appearance, pulse, grimace, activity, and respiration (APGAR) scores, weight at birth and birth defects between babies delivered by Caesarean or delivered vaginally^10^.

### Prevalence of sepsis in infants and maternal antibiotics use during pregnancy

The prevalence of neonatal sepsis was 22.1 % within the general study population with the first 90 days of postnatal life. However, the prevalence of neonatal sepsis was significantly higher in infants whose mothers were treated with antibiotics prenatally than in infants born to untreated mothers (28.8% VRS 9.2%). The relative risk was 4 times higher amongst intrapartum antibiotic treated babies than infants whose mothers were not treated with antibiotics (OR=3.98, *P*<0.001, Cl=95%, K=20.6, DF=1). After adjusting for the influence of method of delivery, the adjusted relative risk was 2.1 (OR=2.091; P= 0.035, Cl=95%, K=4.46, DF=1). The risk of neonatal sepsis was higher in third trimester antibiotic usage and perinatal (24 hours to delivery) antibiotic use (OR=6.64, *P*<0.001, DF=1, K= 61.4) (Table 1). Maternal socio-economic and socio-demographic factors such as occupation (*P* = 0.134), age (*P* = 0.85), gravida (*P* = 0.65), religion did not significantly affect the risk of an infant developing sepsis within the first 90 days of life.

**Table 1:** Prenatal antibiotic use and the risk of neonatal of sepsis in infants within the first ninety days if postnatal life

		Neonatal Sepsis		Total	Significance
		
		Yes	No		
Mother exposed to antibiotic	Yes	78(28.8%)	193(71.2%)	271	K=20.6, **P<0.001**
	No	13(9.2%)	128(90.8%)	141	Df=1, **Rr=3.98**

Perinatal antibiotic exposure	Yes	55(47.8%)	60(52.2%)	115	K=61.4, **P<0.001**
	No	36(12.1%)	261(87.9%)	297	Df=1, **Rr=6.64**

Mother exposed to antibiotic-(Adjusted)^2^	Yes	30(19.0%)	128(81.0%)	158	K=4.46, P=0.035
	No	13(10.1%)	116(89.9%)	129	Df=1, Rr=2.091

Marital Status	Married	65(21.4%)	239(78.6%)	304	K=0.058, P=0.81
	Single	15(22.7%)	51(77.3%)	66	Df=1, Rr=0.925

Age of mother at birth	19 Years and below	8(17.4%)	38(82.6%)	46	K=0.73, P=0.695
	20–30 Years	60(23.0%)	201(77.0%)	261	Df=2
	31 Years and above	22(21.6%)	80(78.4%)	102	

Denomination	Christian	72(21.6%)	261(78.4%)	333	K=0.150, P=0.70
	Muslim	6(25.0%)	18(75%)	24	Df=2
	Traditional	2(25.0%)	6(75.0%)	8	

Gravida	Up to 3	47(22.2%)	165(77.8%)	212	K=0.003, P=0.65
	4 and above	21(21.9%)	75(78.1%)	96	Df=1, Rr=1.017

Occupation	Employed	65(21.9%)	232(78.1%)	126	K=0.256, P=0.88
	Unemployed	7(25.9%)	20(74.1%)	78	Df=2
	Student	10(23.3%)	33(76.7%)	43	

### Prevalence of infant respiratory tract disorders and maternal antibiotics use during pregnancy

The prevalence of respiratory tract disease (RTD) in the intrapartum antibiotic exposed and unexposed group was 18.5 % (50) versus 5.0 % (7). Infants exposed to antibiotics intrapartum were four times more likely to develop respiratory tract disease than unexposed children (OR=4.30, 95 % CI, 1.89 - 9.71, K=13.9, *p*<0.001). The relative risk was still high even after adjusting for the mode of delivery (OR=3.52, K=20.88, *P*<0.001, DF=1). Unlike neonatal sepsis, the period of pregnancy within which the antibiotic was administered to the mother had minimal effects on the relative risk of developing respiratory tract disorders i.e. 3.3, 2.1, 3.4 for each trimester respectively. However, infants who were exposed to antibiotics in all trimesters had six to seven times the relative risk of developing a respiratory tract disorder compared to children who were not treated with antibiotics at all. Maternal socio-demographic factors such as marital status, gravida, age did not influence the relative risk of developing respiratory tract diseases (Table 2).

**Table 2:** Prenatal antibiotic use and risk of neonatal respiratory tract disorder in infants within the first ninety days of postnatal life

		RESPIRATORY TRACT DISEASES		Total	Significance
		
		YES	NO		
Mother exposed to antibiotic	Yes	50(18.5%)	221(81.5%)	271	K=13.9, **P<0.001**
	No	7(5.0%)	134(95.0%)	141	DF=1 **RR=4.30**

Perinatal Antibiotic Exposure	Yes	30(26.1%)	85(73.9%)	115	K=20.88, **P<0.001**
	No	27(26.1%)	270(90.9%)	297	DF=1 **RR=3.52**

Mother exposed to antibiotic- (Adjusted)^2^	Yes	21(13.0%)	140(87.0)	161	K=6.454, **P=0.011**
	No	6(4.5%)	128(95.5%)	134	DF=1, **RR=3.2**

Marital Status	Married	44(14.5%)	260(85.5%)	304	K=0.248, P=0.618
	Single	8(12.1%)	58(87.9%)	66	DF=1, RR=1.22

Age of mother at birth	19 Years and below	4(8.7%)	42(91.3%)	46	K=2.138, P=0.343
	20–30 Years	41(15.7%)	220(84.3%)	261	DF=2
	31 Years and above	12(11.8%)	90(88.2%)	102	

Gravida	1 To 3	26(12.3%)	186(87.7%)	212	K=0.647, P=0.421
	4 and above	15(15.6%)	81(84.4%)	96	DF=1, RR=0.77

Occupation	Employed	38(12.8%) ^B^	259(87.2%)	126	K=6.07, P=0.05
	Student	5(11.6%) ^B^	38(88.4%)	78	DF=2,
	Unemployed	8(29.6%) ^A^	19(70.4%)	27	

### Prevalence of Dermatitis and maternal antibiotics use during pregnancy

A total of sixty-four (64) babies were diagnosed with dermatitis within the first 3 months of post -natal life (Table 3). Out of the 64 babies diagnosed with dermatitis, 52(19.2%) were born to mothers prescribed antibiotics during pregnancy as against 12 (8.5%) in the non-antibiotic treated group. The relative risk (RR) of neonatal dermatitis in prenatal antibiotic treated babies were 2.6 (p= 0.005, Cl=95, K=8.06, DF=1). After adjusting for the influence of method of delivery, neonatal dermatitis was high amongst infants exposed prenatally to antibiotics with a relative risk was 2.3 (P=0.017, Cl=95, K=5.70, DF=1). It was apparent in the study that first trimester antibiotic treatment was associated with higher relative risk of dermatitis than second and third trimesters. Mothers' socio-demographic factors; marital status (P = 0.7), occupation (P = 0.98), and gravida (P = 0.14), were not associated with any significant risk of dermatitis in neonates (Table 3)

**Table 3:** Prenatal antibiotic use and risk of neonatal dermatitis in infants within the first ninety days of postnatal life

		DERMATITIS		Total	
			
		YES	NO		significance
MOTHER EXPOSED TO ANTIBIOTIC	YES	52(19.2%)	219(80.8%)	271	K=8.06, **P=0.005**
	NO	12(8.5%)	129(91.5%)	141	DF=1, **RR=2.6**

PERINATAL ANTIBIOTIC EXPOSURE	YES	24(20.9%)	91(79.1%)	115	K=3.461, P=0.063
	NO	40(13.5%)	257(86.5%)	297	DF=1 RR=1.70

MOTHER EXPOSED TO ANTIBIOTIC- (adjusted)^2^	YES	30(18.9%)	129(81.1%)	159	K=5.70, **P=0.017**
	NO	12(9.0%)	121(91%)	133	DF=1, **RR=2.34**

MARITAL STATUS	MARRIED	52(17.1%)	252(82.9%)	304	K=0.15, p=0.70
	SINGLE	10(15.2%)	56(84.8%)	66	DF=1, RR=1.15

AGE OF MOTHER AT BIRTH	19 YEARS AND BELOW	9(16.1%)	47(83.9%)	56	P=0.451, K=1.59
	20–30 YEARS	43(17.1%)	208(82.9%)	251	DF=1
	31 YEARS AND ABOVE	12(11.8%)	90(88.2%)	102	

GRAVIDA	UP TO 3	28(13.2%)	184(86.8%)	212	K=2.215, P=0.137
	4 AND ABOVE	19(19.8%)	77(80.2%)	96	DF=1

OCCUPATION	EMPLOYED	47(15.8%)	250(84.2%)	297	P=0.986, K=0.027
	STUDENT	7(16.3%)	36(83.7%)	43	DF=2
	UNEMPLOYED	4(14.8%)	23(85.2%)	27	

### Prevalence of Neonatal conjunctivitis and maternal antibiotics use during pregnancy

The prevalence of conjunctivitis within the general study population of neonates was 8.98 % (n=37). The prevalence of conjunctivitis in infants exposed prenatally to antibiotics was 11.8% (n=32) as against 5.0 % (n=7) non exposed group. The relative risk of neonates developing conjunctivitis was 2.56 (P=0.024, Cl=95%, K=5.06). However, after adjusting for the effects of mode of delivery, the risk of neonatal conjunctivitis was not statistically significant 2.0 (P=0.129, Cl=95%, K=2.30). There was no statistically significant association between Mother's age (p = 0.189), religion (p = 0.628), marital status (p= 0.976), gravida (p= 0.538) and occupation (p= 0.729) and the relative risk of neonatal conjunctivitis (Table 4)

**Table 4:** Prenatal antibiotic use and risk of neonatal conjunctivitis in infants within the first ninety days of postnatal life

		NEONATAL CONJUNCTIVITIS		Total	
				
		YES	NO		significance
MOTHER EXPOSED TO ANTIBIOTIC	YES	32(11.8%)	239(88.2%)	271	K=5.06, **P=0.024**
	NO	7(5.0%)	134(95.0%)	141	DF=1, **RR=2.56**

PERINATAL ANTIBIOTIC EXPOSURE	YES	15(13.0%)	100(87.0%)	115	K=2.382, P=0.123
	NO	24(8.1%)	273(91.9%)	297	DF=1, RR=1.61

MOTHER EXPOSED TO ANTIBIOTIC- (adjusted)^2^	YES	16(10.1%)	143(89.9%)	159	K=2.30, P=0.129
	NO	7(5.3%0	126(94.7%)	133	DF=1, RR=2.0

MARITAL STATUS	MARRIED	28(9.2%)	276(90.8)	304	K=0.001, P=0.976
	SINGLE	6(9.1%)	60(90.9%)	66	DF=1, RR=1.01

AGE OF MOTHER AT BIRTH	19 YEARS AND BELOW	6(10.7%)	50(89.3%)	56	P=0.189, K=3.392
	20–30 YEARS	28(11.2%)	223(88.8%)	251	DF=2,
	31 YEARS AND ABOVE	5(4.9%)	97(95.1%)	102	

DENOMINATION	CHRISTIAN	33(9.9%)	300(90.1%)	333	P=0.628, K=0.932
	MUSLIM	2(8.3%)	22(91.7%)	24	DF=2
	TRADITIONAL	0	8	8	

GRAVIDA	1–3	20(9.4%)	192(90.6%)	212	p=0.538, k=0.379
	4 AND ABOVE	7(7.3%)	89(92.7%)	96	DF=1, RR=1.324

OCCUPATION	FARMER	25(8.4%)	272(91.6%)	297	p= 0.729, k=0.633
	STUDENT	5(11.6%)	38(88.4%)	43	DF=2
	UNEMPLOYED	3(11.1%)	24(88.9%)	27	

### Prevalence of Neonatal jaundice and maternal antibiotics use during pregnancy

The prevalence of neonatal jaundice in the study population was 6.8 % (28). The prevalence of jaundice in the antibiotic exposed infants was 23(8.5%) compared to 5(3.5%) in the non-antibiotic exposed group. The relative risk of neonatal jaundice in infants exposed to antibiotic at any point in pregnancy was 2.5 (P = 0.059, 95 % CI, 0.93 - 6.71, K=3.58). After adjusting for mode of delivery, the relative risk was 0.924 (P=0.940, K=0.006). Maternal factors such as gravida, age, marital status did not significantly affect the risk of neonates developing dermatitis (Table 5)

**Table 5:** Prenatal antibiotic use and risk of neonatal Jaundice in infants within the first ninety days of postnatal life

		NEONATAL JAUNDICE		Total	
			
		YES	NO		significance
MOTHER EXPOSED TO ANTIBIOTIC	YES	23(8.5%)	248(91.5%)	271	K=3.58, P=0.059
	NO	5(3.5%)	136(96.5%)	141	DF=1, RR=2.52

PERINATAL ANTIBIOTIC EXPOSURE	YES	11(9.6%)	104(90.4%)	115	K=1.931, P=0.165
	NO	17(5.7%)	280(94.3%)	297	DF=1, RR=1.74

MOTHER EXPOSED TO ANTIBIOTIC-(adjusted)^2^	YES	1(5.3%)	18(94.7%)	19	K=0.006, P=0.940
	NO	16(5.7%)	266(94.3%)	282	DF=1, RR=0.924

MARITAL STATUS	MARRIED	19(6.3%)	285(93.8%)	304	K=0.003, P=0.654
	SINGLE	4(6.1%)	62(93.9%)	66	DF=1 OR=1.03

AGE OF MOTHER AT BIRTH	19 YEARS AND BELOW	3(6.5%)	43(93.5%)	46	K=1.67, P=0.435
	20–30 YEARS	20(7.7%)	241(92.3%)	261	DF=2,
	31 YEARS AND ABOVE	4(3.9%)	98(96.1%)	102	

DENOMINATION	CHRISTIAN	18(5.4%)	315(94.6%)	333	K=5.65, P=0.056
	MUSLIM	1(4.2%)	23(95.8%)	24	DF=2
	TRADITIONAL	2(25.0%)	6(75.0%)	8	

NUMBER OF DELIVERIES	UP TO 3	11(5.2%)	201(94.8%)	212	K=0.00, P=0.994
	4 AND ABOVE	5(5.2%)	91(94.8%)	96	DF=1

OCCUPATION	EMPLOYED	22(7.4%)	275(92.6%)	126	K=1.97, P=0.373
	STUDENT	1(2.6%)	42(97.4%)	78	DF=2
	UNEMPLOYED	1(3.7%)	26(97.7%)	43	

### Mean number of hospital visits and antibiotic exposure

The range of non-scheduled/ non-review postnatal visits was 0 to 6 within the 90 days and a mean of 0.57 ± 0.99 days within the study population. Intrapartum antibiotic treated infants were 3.5 times likely to visit the hospital for treatment within the first 90 days compared to non-treated babies, i.e., 0.73 ± 1.1 days as against 0.20 ± 0.6 days (95 % CI, p<0.001, F = 21.2). After adjusting for method of delivery, the association between antibiotic exposure and mean visits was still significant, 0.53±0.86 days as against 0.20±0.52 days (95 % CI, *p*<0.001, F = 13.92). There was no statistically significant association between the number of visits and other socio-economic factors of the mother (Table 6).

**Table 6:** Mean number of non-review/non-scheduled hospital visits of infants born to mothers treated with antibiotics during pregnancy

MATERNAL PARAMETER		NUMBER OF NON-SCHEDULED VISITS	
		
		(Mean±SD)	Significance
Mother Exposed to Antibiotic	YES	0.73±1.10	p<0.001, F=21.12
	NO	0.26±0.63	

Mother Exposed to Antibiotic (Adjusted)^2^	YES	0.53±0.86	p<0.001, F=13.920
	NO	0.20±0.52	

Perinatal Visits	YES	1.06±1.29	p<0.001, F=42.00
	NO	0.38±0.77	

Marital Status	Married	0.57±1.02	p=0.840, F=0.043
	Single	0.59±0.89	

Age	<19	0.55±0.83	
	20–30	0.59±0.99	p=0.920, F=0.083
	>30	0.54±1.08	

Religion	Christian	0.54±0.98	
	Muslim	0.52±0.85	p=0.244, F=1.416
	Traditional	1.12±0.83	

Gravidae	1 - 3	0.51±0.95	p=0.268, F=1.230
	< 4	0.64±0.99

Employment	YES	0.59±1.02	
	NO	0.38±0.80	p=0.586, F=0.535
	Student	0.53±0.85	

## Discussion

Maternal and neonatal health continue to be a significant developmental challenge for many low-income economies in Sub-Saharan Africa. Poor infant health places a significant financial and time burden on families, who must devote a significant amount of time to caring for a sick child. Infants who are born in poor health are more likely to experience health and social problems later in life than healthy babies. 22. There have been a number of epidemiological studies that suggest antibiotics, one of the most important classes of medicines used in maternal health protection, may be subtly influencing the human body through unspecified mechanisms. 23–25. It has been shown that antibiotics alter maternal microbiome, consequently altering the quality of key microorganisms needed to modulate infant metabolism and immunity 23–25.

In this study, health outcomes of infants (3 months) born to antibiotic exposed mothers at a rural hospital in Ghana were evaluated. Infants treated prenatally with antibiotics had higher prevalence of dermatitis, respiratory disorders, sepsis and subsequently made more non-scheduled visits to the hospital compared to unexposed cohorts. There was a strong association between the prenatal antibiotic use and the risk of developing neonatal sepsis and neonatal respiratory disorder. The relative risk for neonatal sepsis was six to seven times higher when antibiotics were administered within 24 hours of delivery. There was also a moderate association between neonatal dermatitis and maternal prenatal antibiotic use. Similar reports and trends have been noted in similar studies in other parts of the world by other authors. It has been reported by other authors that intrapartum antibiotic is a significant risk factor for not only asthma and other allergic diseases but infectious diseases in later life 26,27.

The development of the infant immune system and metabolism depends partly on the quality of microbiota inherited from the mother 28. The quality of maternal microbiota correlates well with infant health 15–17. Alteration or disturbance in the infant acquisition of maternal microbiome could result in the occurrence of both infectious, allergic and immune disorder 29–30. The same reason could account for the difference in health due to mode of delivery. It is known that each microbial community controls the growth and replication of other species, thus ensuring a dynamic equilibrium and symbiosis 31. Antibiotic use generally disturbs the microbial ecology either by complete deletion or reduction in levels of susceptible microbes or by selective proliferation of resistant species^31^.

Epigenetic factors have been known to affect the expression of transcription genes needed to regulate the population of T cells. Bacterial metabolites and short fatty acid chains have been implicated in modification of histone bodies 32. This affects the wrapping of DNA and this could affect how tightly or loosely a section of the DNA is available for transcription. Changes in T cell populations could be a reason for the observed differences in health outcomes between neonates who had altered microbiota and those who were not 29. This confirms the phenomenon seen in other animal models in which restoration of a normal microbiome in experimented mice does not restore a balanced immune system. It is worth noting that these epigenetic changes occur during foetal and early life and may be irreversible once established. Some studies designed to look at antibiotic exposure either intrapartum or early childhood and risk of allergic respiratory tract disease have yielded very conflicting results. For instance, Leickly (2003) in a study found no association between antibiotic use and allergic respiratory tract diseases, but other studies by Droste et al. (2000) and Pistiner et al. (2008) found higher risk of respiratory diseases among infants exposed to antibiotics or who were delivered by caesarean birth 33–35.

## Conclusion

Infants prenatally treated with antibiotics had higher risk of developing neonatal sepsis, neonatal respiratory disorder, and dermatitis. The infants were 3.5 times likely to visit the hospital for non-scheduled or non-review treatment.

## Data Availability

All data for the study will be made available upon request to the corresponding author.
